# Lactate and lactylation in macrophage metabolic reprogramming: current progress and outstanding issues

**DOI:** 10.3389/fimmu.2024.1395786

**Published:** 2024-05-21

**Authors:** Bangjun Xu, Yi Liu, Ning Li, Qing Geng

**Affiliations:** Department of Thoracic Surgery, Renmin Hospital of Wuhan University, Wuhan, China

**Keywords:** lactate, lactylation, Post-translational modification (PTM), macrophage, metabolic reprogramming

## Abstract

It is commonly known that different macrophage phenotypes play specific roles in different pathophysiological processes. In recent years, many studies have linked the phenotypes of macrophages to their characteristics in different metabolic pathways, suggesting that macrophages can perform different functions through metabolic reprogramming. It is now gradually recognized that lactate, previously overlooked as a byproduct of glycolytic metabolism, acts as a signaling molecule in regulating multiple biological processes, including immunological responses and metabolism. Recently, lactate has been found to mediate epigenetic changes in macrophages through a newfound lactylation modification, thereby regulating their phenotypic transformation. This novel finding highlights the significant role of lactate metabolism in macrophage function. In this review, we summarize the features of relevant metabolic reprogramming in macrophages and the role of lactate metabolism therein. We also review the progress of research on the regulation of macrophage metabolic reprogramming by lactylation through epigenetic mechanisms.

## 1 Introduction

Traditionally, both tissue-resident and recruited macrophages polarize into two simplified distinct phenotypes stimulated by microenvironmental factors in pathological conditions: the classically activated phenotype (M1) and the alternatively activated phenotype (M2) (1). M1 macrophages, which are typically activated by lipopolysaccharide (LPS) or Th1 cytokines (e.g., IFN-γ, GM-CSF), are pro-inflammatory and produce high levels of pro-inflammatory cytokines (2). In contrast, M2-type macrophages can be polarized by Th2 cytokines (e.g., IL-4, IL-13, IL-10) to possess anti-inflammatory properties. IL-4 and IL-13 activate STAT6 via IL-4Rα, whereas IL-10 activates STAT3 via IL-10R, leading to the polarization of M2 macrophages (3). M2 macrophages exhibit anti-inflammatory effects through the expression of various anti-inflammatory molecules and endocytosis receptors, such as scavenger receptors (4). Besides, M2 macrophages generate profibrotic and pro-repair factors like PDGF, VEGF, THBS1, and TGF-β1. They also play a role in inducing the epithelial-mesenchymal transition (EMT) in alveolar epithelial cells, as well as the fibroblast-to-myofibroblast transition (FMT), contributing to fibrosis and tissue repair (5, 6). Beyond the traditional M1 and M2 macrophage phenotypes, macrophages recruited into the tumor microenvironment (TME) during tumorigenesis and progression are referred to as tumor-associated macrophages (TAM). In the early stages of tumorigenesis, TAM typically exhibits an immune-promoting phenotype with pro-inflammatory and anti-tumor effects, resembling the M1 phenotype (7). However, as tumors progress, factors like an acidic environment and hypoxia in the TME can induce a metabolic reprogramming of TAM towards an immunosuppressive phenotype akin to the M2 phenotype. This shift promotes tumor growth, angiogenesis, and metastasis, which is correlated with a poorer prognosis (8–10).

In macrophages, metabolic reprogramming refers to adaptive changes in the cell’s energy metabolism during its differentiation and activation (11). Macrophages with different phenotypes have some degree of metabolic heterogeneity in pathways such as glycolysis, the pentose phosphate pathway (PPP), oxidative phosphorylation (OXPHOS), the tricarboxylic acid cycle (TCA cycle), fatty acid synthesis (FAS), arginine metabolism, and glutamine metabolism. This heterogeneity of metabolic profiles correlates with the heterogeneity of macrophage phenotypes (12). Macrophages display plasticity in both their metabolic and functional characteristics, with their metabolic processes being modulated by the surrounding microenvironment, subsequently influencing their activation and polarization states (13). This phenotypic plasticity is emerging as a novel therapeutic tool for the treatment of tumors and chronic inflammatory diseases such as atherosclerosis and rheumatoid arthritis, by regulating macrophage signaling and metabolic pathways as well as reprogramming the macrophage phenotype (14–17). However, different phenotypes of macrophages have varying degrees of plasticity. M2 macrophages are highly plastic, enabling them to easily repolarize to the M1 phenotype. Meanwhile, M1 macrophages are unable to repolarize to the M2 phenotype due to the damaging effect of NO produced by arginine metabolism on the mitochondrial oxidative phosphorylation process. Consequently, the plasticity of macrophage phenotypes is intricately linked to their metabolic features (18).

Lactate is the end product of glycolysis, and it has long been recognized as a waste product of metabolism (19). However, evidence is now mounting that lactate has multiple biological functions. Lactate can act as a signaling molecule to regulate various processes such as metabolism, immune response, and intercellular communication (20). Lactylation is a novel histone post-translational modification first discovered and proposed by Zhang D et al. in 2019. In macrophages, Zhang D et al. found that the intracellular lactate accumulated both endogenously via the glycolytic pathway and exogenously by taking up from the extracellular milieu, can lactylate histone lysine through an endogenous “lactate clock” mechanism, which regulates the transcriptional expression of related genes that transform macrophage phenotypes (21). As a newly discovered post-translational modification, lactylation has been found in both histones and non-histone proteins. Histone modifications can regulate gene transcription and expression by altering chromatin structure, while non-histone modifications on proteins including transcription factors and metabolic enzymes can regulate their functions, such as protein stability, activity, interactions, and localization (22, 23). It has been known that covalent histone modifications like acetylation and methylation can achieve chromatin remodeling by affecting histone-DNA, histone-histone, and histone-chaperone interactions (24). Alterations in the relaxed/condensed state of chromatin can affect the binding of transcription factors to promoters, thereby affecting gene transcription and expression (25). In Zhang D et al.’s study, the ChIP-seq data showed that both lactylation and acetylation at the histone H3K18 site were enriched in the promoter region. In addition, they demonstrated that the H3K18la modification at the promoter during M1 polarization can directly promote the transcription of homeostatic genes (21). Nonetheless, further evidence is needed to support the molecular mechanism through which lactylation regulates the transcription of target genes.

There are two different isomers of lactate: L-lactate and D-lactate. These correspond to two lactylation pathways, L-lactylation (direct lactylation) and D-lactylation (indirect lactylation). L-lactate and its lactylation modification process are primarily utilized to regulate physiological and pathological processes such as signal transduction (26). Therefore, unless otherwise specified, “lactate” herein generally refers to L-lactate. D-lactate is mainly produced from methylglyoxal (MGO), a byproduct of glycolysis, via the glyoxalase pathway (27). In 2020, Gaffney DO et al. found that lactoylglutathione (LGSH), which is not able to produce D-lactate in the absence of glyoxalase 2 (GLO2), indirectly modifies non-histone proteins, such as glycolytic metabolic enzymes, through non-enzymatic lysine lactylation, thereby regulating metabolic pathways such as glycolysis through negative feedback mechanism (26, 28). D-lactate can also act as a signaling molecule in the regulation of macrophage polarization, but the direction of macrophage polarization depends on the signaling pathways regulated by L-lactate (29, 30). Studies also demonstrated that HDAC1–3 serve as major cellular delactylases, which reversibly and dynamically regulate histone lactylation. In addition, the delactylase activities of HDAC1 and HDAC3 are site-specific (26).

In short, there is increasing evidence that lactate, an important metabolite of macrophages, can play a role in the metabolic reprogramming process of macrophages through lactylation modification to regulate macrophage phenotypes and help them adapt to different pathophysiological environments. In this review, we summarize the characteristics of relevant metabolic reprogramming in macrophages as well as the role of lactate metabolism therein. Furthermore, we discuss the molecular mechanisms by which lactylation regulates macrophage metabolic reprogramming as well as the role of lactylation in the different pathophysiological processes in which macrophages are involved.

## 2 Relevant metabolic reprogramming of macrophage

### 2.1 Glycolysis and oxidative phosphorylation

Glycolysis is commonly recognized as an important metabolic pathway for cells to produce lactate and energy from glucose under hypoxic conditions, but pro-inflammatory macrophages and tumor cells prefer to rely on glycolysis in normoxia (12, 31). Despite being less efficient than OXPHOS in terms of the amount of ATP yielded, producing only 2 molecules of ATP per glucose molecule, glycolysis excels in producing ATP rapidly (32). Generally speaking, macrophage polarization to M1/M2 phenotype requires glucose metabolic reprogramming: M1 macrophages exhibit enhanced aerobic glycolysis and diminished OXPHOS to meet short-term energy demands of acute inflammation at the early stage of an infection, while M2 macrophages display enhanced mitochondrial OXPHOS (33–35). Studies have demonstrated that inhibiting glycolytic processes prevents macrophage polarization to the M1 phenotype, leading to the attenuation of LPS-induced inflammatory responses and reduced acute lung injury (ALI) in mice (36, 37). Hypoxia-inducible factor-1α (HIF-1α) contributes to the regulation of glycolysis as well as the induction of pro-inflammatory gene expression, and it plays a crucial role in macrophage activation and polarization to the M1 phenotype (38, 39). Moreover, Pyruvate Kinase M2 (PKM2), a key enzyme promoting the Warburg effect, serves as an important regulator of macrophage polarization, glycolytic metabolic reprogramming, and IL-1β production (36, 40). The HIF-1α/PKM2 axis plays a crucial role in driving the M1 phenotype transformation and pro-inflammatory activities, regulating target genes like glucose transporter protein 1 (GLUT1), glycolytic enzymes such as lactate dehydrogenase A (LDHA) and pyruvate dehydrogenase kinase 1 (PDK1), as well as pro-inflammatory factors such as IL-1β (38, 41). Meanwhile, PKM2 also promotes the secretion of HMGB1 protein, which mediates inflammatory responses in activated macrophages (42). Activation of PKM2 through DASA-58 and TEPP-46 leads to PKM2 activation via tetramer formation, suppressing the Warburg effect and lactate production without nuclear translocation. The above phenomenon is known as “the PKM2 paradox in the Warburg effect”. This process resulted in the downregulation of M1 phenotypic gene expression in macrophages regulated by the HIF-1α-PKM2 interaction, attenuating the LPS-induced M1 macrophage phenotype and promoting a shift towards typical M2 features (40, 43). In conclusion, the Warburg effect is critical for the polarization and maintenance of the M1 macrophage phenotype (44).

**Figure 1:**
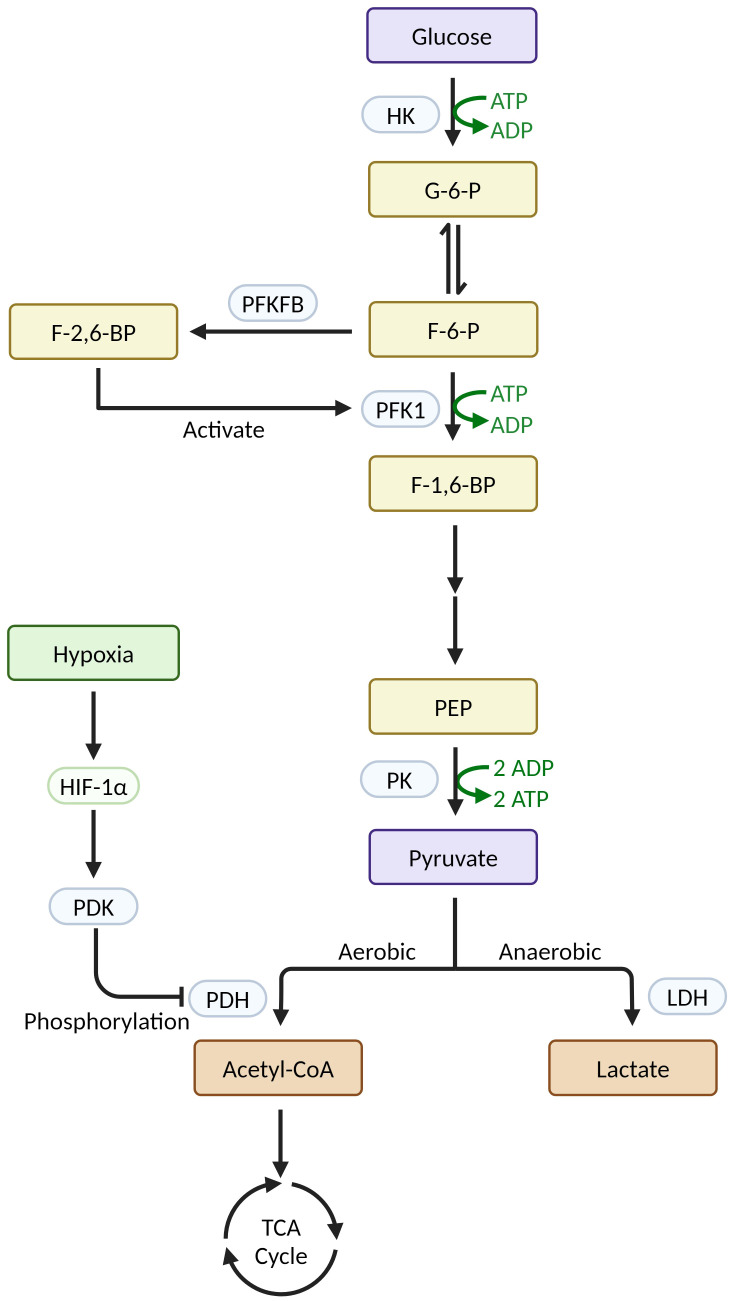
Relevant enzymes in macrophage glycolysis and their regulatory mechanisms (Created with BioRender.com).

During glycolytic metabolism in macrophages, a series of glycolytic enzymes play crucial roles in regulating metabolic reprogramming (**Figure 1**). The first enzyme that limits the rate of glycolysis is hexokinase (HK), which catalyzes the transformation of glucose into glucose-6-phosphate (G-6-P). In sepsis, Yuan Y et al. showed that the transcription factor KLF14 suppresses immune function by inhibiting the transcription of HK2, decreasing glycolysis, and causing M1-type macrophages to secrete inflammatory factors less (45). In their study on autoimmune thyroid disease, Cai T et al. found that HK3 knockdown or knockout reduced the proportion of M1 macrophages in both *in vitro* and *in vivo* experiments, supporting the idea that HK3 induces macrophage polarization toward the M1 phenotype through metabolic reprogramming (46). The key rate-limiting step in glycolysis is the transformation of F-6-P to F-1,6-BP, which is catalyzed by phosphofructokinase 1 (PFK1). Fructose-6-phosphate-2-kinase/fructose-2,6-bisphosphatase (PFKFB) catalyzes the transformation of F-6-P to F-2,6-BP, which is the strongest activator of PFK1 (47). Schilperoort M et al. found that the PFKFB2-mediated glycolytic pathway is rapidly activated in macrophages after phagocytosis and that the lactate it produces promotes the expression of the efferocytosis receptors MerTK and LRP1 via calcium signaling, thereby driving sustained phagocytosis (48). Mager CE et al. found that MKP-1 defects mediate increased PFKFB3 expression via the p38 MAPK pathway in both an E. coli-infected mouse model of sepsis and an LPS-stimulated macrophage model, leading to upregulation of macrophage glycolysis and the development of sepsis (49). The rate-limiting enzyme of glycolysis, pyruvate kinase (PK), catalyzes the conversion of phosphoenolpyruvate (PEP) to pyruvate. Its isoform, PKM2, is a crucial enzyme that stimulates the inflammatory response and Warburg effect in macrophages (42). PDK1, an important regulator of glucose metabolism, inactivates pyruvate dehydrogenase (PDH) by phosphorylation, thereby preventing the production of acetyl-CoA from pyruvate and facilitating lactate production via the glycolytic pathway (12). In a study on atherosclerosis, Forteza MJ et al. found that the promotion of vascular inflammation by the PDK/PDH axis was associated with M1 macrophage polarization (50). Furthermore, Semba H et al. found that PDK1 promotes both M1 macrophage polarization and macrophage migration under mild hypoxia by inducing glycolysis via the HIF-1α-PDK1 axis (51).

There is ongoing debate concerning the function of glycolysis in M2 macrophages. It was previously believed that M2 macrophages had lower glycolysis levels compared to M1 macrophages since they primarily relied on OXPHOS for glucose metabolism (52). However, recent studies have revealed a potentially significant role of glycolysis in M2 macrophage activation through the use of 2-DG, a glycolysis inhibitor. Several studies have found that 2-DG inhibits glycolysis and OXPHOS, impacting M2 macrophage activation (18, 53). A study by Covarrubias AJ et al. discovered an increase in glucose uptake in IL-4-treated BMDM, which correlated with an AKT-dependent rise in glycolysis and oxidative metabolism. The researchers found that similar to the β-oxidation inhibitor Etomoxir, 2-DG reduced the expression of certain M2 genes induced by IL-4 (e.g., Arg1, Retnla, Mgl2), suggesting that glycolysis is required for M2 macrophage activation. However, their study also revealed that IL-4 activates ATP citrate lyase (Acly) in a phosphorylated manner via the AKT-mTORC1 pathway, independently of the classical JAK-STAT6 pathway. Acly is a key enzyme for acetyl CoA synthesis, and its expression can be induced by increasing histone acetylation of the aforementioned M2 genes (also known as AKT-dependent M2 genes). These findings imply that glycolysis is not the only pathway for M2 macrophage activation (54, 55). Subsequently, Huang SC et al. demonstrated that IL-4 enhanced glycolysis in BMDM by increasing IRF4 expression through the mTORC2 pathway, thereby activating M2 macrophages. In their research, they found that 2-DG significantly inhibited the expression of M2-type activation markers such as RELM-α and PD-L2, and led to a decrease in the glycolytic indicators like extracellular acidification rate (ECAR) and glycolytic reserve (GR) (56). However, Wang F et al. showed that the 2-DG used to block glycolysis in the aforementioned study had a possible off-target effect: higher doses of 2-DG not only inhibited glycolysis and OXPHOS but also suppressed M2-type polarization of macrophages by reducing intracellular ATP concentration as well as down-regulating JAK-STAT6 signaling. It was found that the activation and polarization of M2 macrophages through the JAK-STAT6 pathway required a threshold level of intracellular ATP, which could be achieved by accumulating via the glycolysis/OXPHOS pathway. Therefore, glycolysis was not necessary for the activation of M2 macrophages when OXPHOS functioned normally. In their study, they treated IL-4-stimulated BMDM by glucose consumption or by using galactose instead of glucose and examined the expression of M2-type activation markers. The results showed that glycolytic inhibition did not affect BMDM activation when OXPHOS hadn’t been damaged in BMDM (57). In conclusion, the specific role of glycolysis in activating M2 macrophages requires further investigation.

### 2.2 Arginine metabolism

Arginine undergoes complex metabolism in the body, serving as a precursor for various important biological compounds including nitric oxide (NO), citrulline, ornithine, urea, proline, glutamate, creatine, and polyamines (58). Different phenotypes of macrophages are characterized by distinct arginine metabolism. M1 macrophages express iNOS, which converts arginine to NO and citrulline. NO, along with its reactive nitrogen species derivatives, play diverse roles in macrophages: (1) They enable macrophages to exert proinflammatory, antibacterial, and cytotoxic effects (59, 60); (2) They inactivate iron-sulfur-containing complexes (mainly complexes I and II) in the mitochondrial electron transport chain (ETC), attenuates OXPHOS and prevents repolarization of M1-type macrophages to M2-type (18); (3) NO can participate in the metabolic reprogramming of the macrophage TCA cycle: NO’s suppression of ACO2 leads to TCA cycle disruption. Additionally, NO can inhibit PDH and diminish its carbon flux in ways that are independent of the HIF-1-PDK1-PDH axis. Since glutaminolysis is a major anaplerotic pathway in macrophages, the above effects lead to increased uptake and utilization of glutamine by macrophages to compensate for the inhibitory effects of NO on ACO2 and PDH (61, 62); (4) NO biphasically regulates iNOS expression in LPS-stimulated mouse macrophages via NF-κB. That effect depends on the local concentration of NO and up-/down-regulates the expression of pro-inflammatory proteins in the body, including iNOS and IL-6 (63). M2 macrophages express Arg1, which catabolizes arginine into ornithine and urea, in contrast to M1 macrophages. Ornithine can be catalyzed by ornithine decarboxylase (ODC) to synthesize polyamines and by ornithine aminotransferase (OAT) to synthesize proline (58). Meanwhile, the regulation of humoral immunity, the antiparasitic response, the allergic response, fibrosis, and wound healing is mediated by Arg1 and its catalytic downstream products (60, 64). Due to the pro-inflammatory and cytotoxic nature of NO, iNOS competes with Arg1 for the catabolic pathway of arginine to balance immune function and its damaging effects (60). Various pathogens, including Leishmania, Schistosoma mansoni, Toxoplasma gondii, and Mycobacterium TB, can compete with iNOS for the catabolization of arginine by upregulating the expression of Arg1, thereby reducing NO production to facilitate infections (44, 65–67).

Since macrophages have dichotomies similar to the iNOS/Arg1 dichotomy in arginine metabolism in many of the major metabolic pathways, the classification of M1/M2 phenotypes has been widely used in the literature as a classic and simple dichotomy. However, the phenotypic classification of different activated macrophages based solely on traits related to arginine metabolism is an oversimplified way of typing. The boundaries of the phenotypic classification of macrophages in question are not yet completely clear, and the distinction of biological behaviors of macrophages that have different phenotypes remains to be investigated (60).

## 3 The linkage between lactate metabolism and macrophage metabolic reprogramming

The Warburg effect is present in a variety of cells, including M1 macrophages and tumor cells, which metabolize glucose to lactate under aerobic conditions. There are several features of the Warburg effect: (1) Aerobic glycolysis rapidly synthesizes ATP in the cytoplasm to meet short-term energy demands; (2) Intermediate products of aerobic glycolysis facilitate the biosynthesis of nucleotides, amino acids, and lipids; (3) Reduced production of reactive oxygen species (ROS) from mitochondrial OXPHOS lessens the harm that oxidative stress causes (19, 68, 69). Thus, to fit their survival milieu, M1 macrophages and tumor cells exhibit the Warburg effect as a result of metabolic reprogramming.

To perform its roles as a major energy source for mitochondrial respiration, a key glycolytic precursor, and a signaling molecule, lactate shuttles between cells and between compartments within the cell. This process connects the aerobic and glycolytic metabolic pathways. Cell-cell lactate shuttles are ubiquitous across various cells, tissues, and organs in the body, while intracellular lactate shuttles include cytoplasmic-mitochondrial exchange and cytoplasmic-peroxisomal exchange (70, 71). Mediated by transport proteins and receptors, the lactate shuttle involves several monocarboxylate transporters (MCTs), the two most extensively researched of which are MCT1 and MCT4: MCT1 is widely expressed in tissues and is involved in lactate uptake, while MCT4 is predominantly expressed in tissues with high glycolysis and is involved in lactate efflux (72, 73). However, the direction of lactate transport by MCTs *in vivo* depends on the lactate and proton concentration gradients (74).

**Figure 2:**
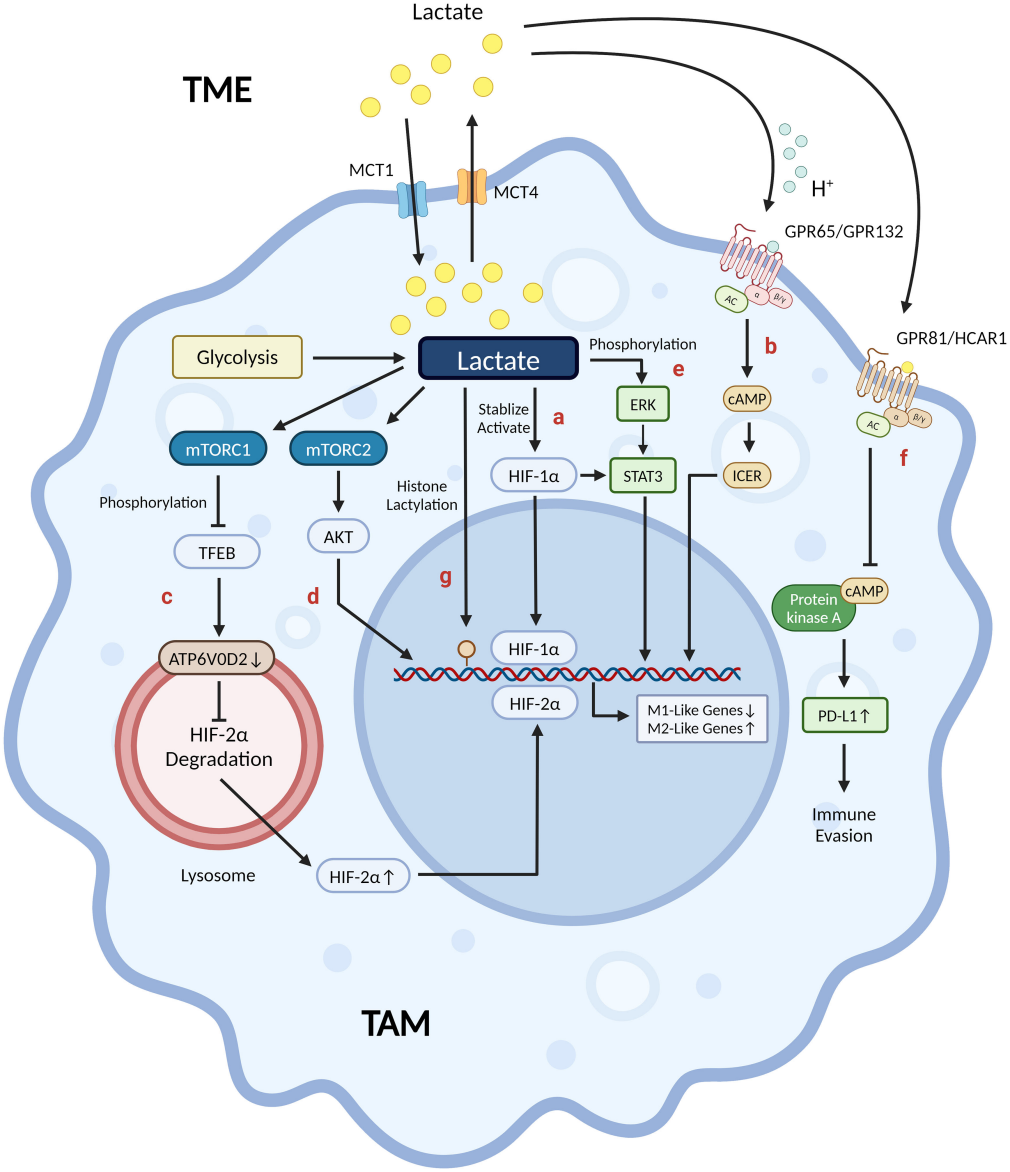
Lactate’s mechanisms in shaping tumor-associated macrophages into an M2-like phenotype. **(A)** Lactate promotes Arg-1 and VEGF expression through stabilization/activation of HIF-1α. **(B)** Proton-sensing GPR65/GPR132 activated by acidic TME from extracellular lactate induces M2-like gene expression via the cAMP-ICER pathway. **(C)** Lactate-activated mTORC1 negatively regulates TFEB, which reduces HIF-2α degradation by down-regulating ATP6V0D2 expression on the surface of the lysosome, thereby regulating immunosuppressive and pro-angiogenic M2-like gene expression. **(D)** Lactate-activated mTORC2-AKT pathway induces TAM to a pro-invasive M2-like phenotype. **(E)** Lactate activates the ERK-STAT3 pathway by phosphorylating ERK, which in turn induces anti-inflammatory and pro-angiogenic M2-like phenotypes. **(F)** GPR81/HCAR1 is activated by physiological concentrations of extracellular lactic acid, which induces PD-L1 expression via down-regulation of the cAMP-PKA pathway, thereby promoting tumor immune evasion. **(G)** Lactate promotes histone lactylation modification (yellow circle) at the promoter H3K18 site of Arg-1, VEGF and other M2-like genes in TAM to up-regulate their expression, thereby inducing an M2-like phenotype in TAM. (Created with BioRender.com).

Previous studies have shown that lactate, as a core molecule, plays a crucial role in regulating macrophage metabolic reprogramming. It has been found to inhibit the activation of M1 pro-inflammatory macrophages while promoting the polarization of M2 macrophages towards an anti-inflammatory and pro-angiogenic phenotype. These effects are mediated through various mechanisms, such as post-translational modification of histones and modulation of signaling pathways (75, 76): (1) Lactate is metabolized to pyruvate via LDH1, which stabilizes HIF-1α via inhibition of prolyl hydroxylase. HIF-1α promotes the expression of Arg-1 and VEGF in TAM in an IL-4/IL-13-independent manner, thereby inducing TAM to an M2-like phenotype. However, HIF-1α does not induce Arg-1 or VEGF during macrophage M1 polarization because HIF-1α protein expression is induced early and HIF-1α binds to the promoters of glycolytic genes, not Arg-1 or VEGF (21, 76–78) (**Figure 2A**). (2) Proton-sensing G protein-coupled receptors GPR132 and GPR65, which are highly expressed on the surface of macrophages, are activated by acidic TME from extracellular lactate. This activation triggers an intracellular signaling cascade that induces the expression of anti-inflammatory and pro-angiogenic genes in macrophages via the cAMP-ICER pathway, thereby promoting macrophage polarization to the M2 phenotype (79–83) (**Figure 2B**). (3) Lactate negatively regulates TFEB through activation of mTORC1, which in turn decreases lysosomal degradation of HIF-2α by down-regulating macrophage ATP6V0d2 expression, thereby increasing macrophage anti-inflammatory and pro-angiogenic gene expression (84) (**Figure 2C**). (4) Lactate promotes tumor invasion by activating the mTORC2-AKT signaling pathway and promoting TAM polarization towards the M2 phenotype (85) (**Figure 2D**). (5) Lactate increases phosphorylation activation of the ERK/STAT3 pathway in macrophages and promotes macrophage M2 phenotype polarization, exerting anti-inflammatory and pro-angiogenic effects (86) (**Figure 2E**). (6) Macrophage G protein-coupled receptor GPR81 (HCAR1) is activated by physiological concentrations of lactate to downregulate the cAMP-PKA signaling pathway, which can mediate immunosuppression, promote angiogenesis, and other processes in tumor tissues (72, 87, 88) (**Figure 2F**). In addition, GPR81 is predominantly located in cell membranes but has also been identified in organelles, indicating its involvement in lactate transport between cell membranes and intracellular compartments (87). In a study concerning the mechanisms regulating the immunosuppressive macrophage phenotype in the pre-metastatic niche, Morrissey SM et al. found that lactate is involved in the regulation of the “non-classical M1” phenotype of macrophages. Tumor-derived exosomes (TDEs) activate two independent pathways that enhance glycolysis via the NF-κB pathway: (1) Activated HIF-1α upregulates GLUT-1 expression, which increases glucose uptake into the macrophage; (2) Activated NOS2 increases NO production, which diverts pyruvate to the lactate pathway by inhibiting OXPHOS. The aforementioned mechanisms cause macrophages to produce more lactate due to increased glycolysis, which can then stimulate PD-L1 expression by activating NF-κB, hence enhancing PD-L1-mediated immunosuppression (89). In contrast, Han S et al. found in a hepatocellular carcinoma model that D-lactate polarized M2 TAM to M1 phenotype and remodeled the immunosuppressive TME in hepatocellular carcinoma through inhibition of the PI3K-AKT pathway and activation of the NF-κB pathway (29).

## 4 Lactylation modifications are involved in regulating macrophage phenotype

**Figure 3:**
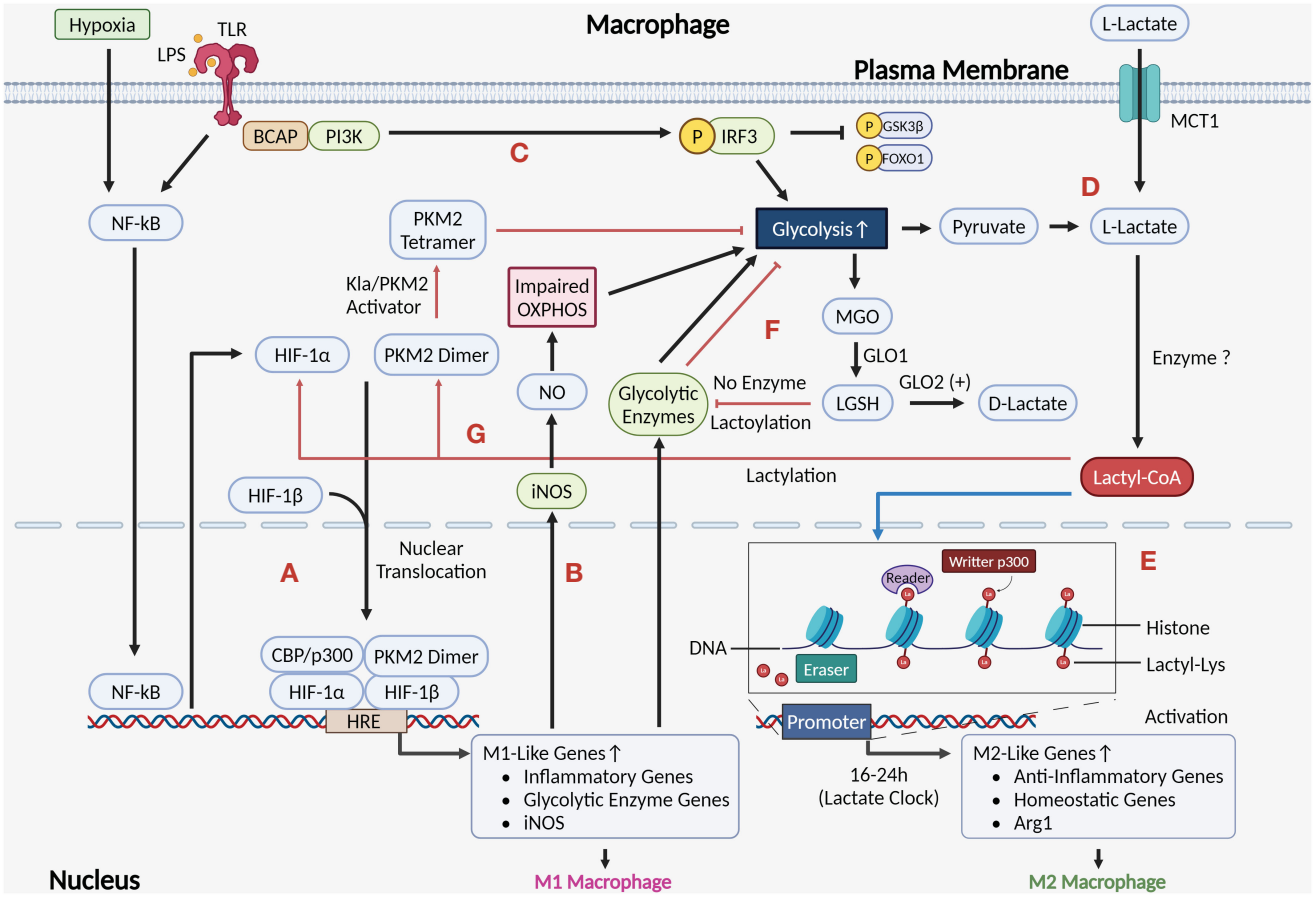
Role of the Warburg effect as well as histone and non-histone lactylation mediated by lactate in the regulation of macrophage phenotype. **(A)** HIF-1α induced by activation of NF-κB signaling after exposure to pro-inflammatory stimuli such as LPS and hypoxia can undergo nuclear translocation and form a complex by interacting with HIF-1β and PKM2 dimer, which also undergo nuclear translocation. This complex induces the expression of M1-like genes by acting on the HRE at the promoters of the target genes, polarizing macrophages to the M1 phenotype. **(B)** Activation of M1 macrophages upregulates the expression of iNOS, which metabolizes arginine to NO. Accumulation of NO damages OXPHOS, leading to enhanced glycolysis in M1 macrophages. **(C)** LPS-recognized TLR activates PI3K-AKT via BCAP, which reduces inflammation by phosphorylating and inhibiting downstream molecules GSK3β and FOXO1, as well as enhances glycolysis to produce lactate. **(D)** Lactate (L-lactate) in the cytoplasm can be either endogenous lactate that accumulates via macrophage glycolysis production or exogenous lactate that is taken up from the extracellular environment via MCT1 at the plasma membrane. **(E)** Histone lactylation is shown by the blue arrow. Lactate in M1 macrophages can be catalyzed by currently unidentified enzymes to generate Lactyl-CoA, which initiates an endogenous “lactate clock” after entering the nucleus, and M2 signature genes modified by lactylation are activated for expression, resulting in cells exhibiting M2-like features. **(F)** The glycolytic byproduct MGO binds to glutathione via GLO1 to form LGSH. LGSH cannot produce D-lactate in the absence of GLO2, but rather indirectly modifies glycolytic enzymes through non-enzymatic lysine lactoylation, which negatively regulates glycolysis by inhibiting glycolytic enzymes (as shown by the red arrows). **(G)** The red arrows show the processes in which non-histone lactylation is involved in regulation. Lactate accumulation from M1 macrophage glycolysis can result in the lactylation of PKM2, which activates the PKM2 dimer to a tetrameric form and exerts an inhibitory effect on macrophage glycolysis. In addition, the stability of HIF-1α in M1 macrophages may also be regulated by lactylation modification. (Created with BioRender.com).

Zhang D et al., 2019 discovered that M1 macrophages have an endogenous “lactate clock”, which regulates the transition of M1 macrophages to exhibit M2-type characteristics during the late stage of polarization through histone lactylation. They propose a regulatory mechanism whereby both endogenous lactate produced by aerobic glycolysis (Warburg effect) and exogenous lactate taken up by MCT in M1 macrophages can be enzymatically reacted to produce lactoyl coenzyme A, which adds a lactoyl moiety to the lysine tail of histones in a p53-dependent manner mediated by the acetyltransferase p300 (21). Although Patel R et al. predicted acetyl-CoA synthetase as a potential enzyme by using molecular docking and molecular dynamics (MD) simulations, there is still no direct evidence for the existence of lactyl-CoA synthetase or transferase that activates lactate to lactyl-CoA in mammals (90). This histone lactylation modification increases in a time-dependent manner and is predominantly present at the promoters of M2-type characteristic wound repair genes such as Arg-1. After 16–24h, these H3K18la-modified M2 genes were activated for expression. M1 macrophages undergo an iNOS→Arg1 expression switch, transitioning to the M2 phenotype to repair tissue damage caused by infection, etc. (21, 91, 92) (**Figures 3D, E**). Subsequent studies have observed similar phenomena in pulmonary fibrosis, post-myocardial infarction repair, and intestinal inflammatory modulation (93–95). In a subsequent research on TAM, Noe JT et al. proposed that early M1 macrophages with high glycolytic/low TCA activity could be modified by histone lactylation using lactate produced by glycolysis as the initiation of the M1→M2 phenotypic transition. Later, with the decrease of glucose and increase of lactate in the microenvironment, M2 macrophages undergo metabolic reprogramming leading to high TCA activity. This process promotes M2-type gene expression, immunosuppression, and tumor progression by converting lactate to pyruvate for entering into the TCA cycle as well as producing acetyl-coA to promote histone acetylation-dependent gene expression (57, 96).

However, the above findings remain controversial. Further research by Dichtl S et al. showed that in LPS-activated macrophages, histone lactylation did not correlate with the activation of M2 macrophages or the expression of genes associated with M2 activation, such as Mrc1, Retnla, and PD-L2. LPS-induced upregulation of Arg-1 expression was dependent on the autocrine/paracrine IL-6 and its downstream activated STAT3 signaling, without involving histone lactylation upregulation. The primary cause of the intracellular accumulation of lactate and the upregulation of lactylation in M1 macrophages is the detrimental effect of NO generated by arginine metabolism on mitochondrial OXPHOS (97). This implies that histone lactylation may not be the exclusive regulatory pathway for genes involved in M2 activation. Furthermore, Van den Bossche J et al. have shown in a previous study that the impairing effect of NO on mitochondrial OXPHOS function inhibits the phenotypic repolarization process M1→M2 (18) (**Figure 3B**). Therefore, there may be two mechanisms for the M1→M2 phenotype transition described previously: (1) Induced by histone lactylation or other pathways, Arg-1 competes with iNOS for arginine catabolism, which reduces NO production and attenuates the degree of damage by OXPHOS, allowing M1 macrophages to repolarize to the M2 phenotype to a certain extent; (2) Lactate produced by M1 macrophages via glycolysis changes the extracellular microenvironment, prompting polarization of recruited circulating monocytes to the M2 phenotype. In sum, more research is needed to determine the precise function lactylation plays in the macrophage M1→M2 phenotypic change.

As a newly identified toll-like receptor (TLR) signaling adapter, the function of the B-cell adapter for PI3K (BCAP) in controlling macrophage transformation into an M2 repair phenotype is confirmed by the study of Irizarry-Caro RA et al (98). In macrophages, recognition of LPS by TLR4 activates NF-κB, which ultimately generates pro-inflammatory factors and improves phagocytosis (99, 100). TLR also activates PI3K-AKT via BCAP and activated AKT contributes to the transformation of macrophages into an anti-inflammatory repair phenotype via two pathways: (1) PI3K-AKT-GSK3β-FOXO1 pathway: phosphorylation of AKT inhibits FOXO1 and GSK3β, its downstream molecules, thus downregulating inflammation; (2) Aerobic glycolysis (the Warburg effect) and histone lactylation: AKT increases lactate production by enhancing aerobic glycolysis. Lactate accumulation causes histone lactylation, which upregulates the expression of wound repair genes (93) (**Figure 3C**). The significance of this study lies in: (1) suggesting that BCAP has a crucial switch-like role in promoting macrophages to switch to the M2 repair phenotype; (2) explaining a regulatory mechanism of the Warburg effect in macrophages at the molecular level; (3) providing a possible drug target for the treatment of diseases caused by over-activation of the macrophage pro-inflammatory phenotype.

Non-histone lactylation modification also plays an important role in regulating macrophage phenotype. In particular, non-histone lactylation has been shown to metabolically reprogram the immune phenotype of macrophages by modulating the activity of enzymes involved in aerobic glycolysis. Gaffney DO et al. discovered non-enzymatic lysine lactoylation, which negatively regulates glycolysis by modifying glycolytic enzymes, inhibiting enzyme activity, and reducing the level of glycolytic metabolites. LGSH mediates this lactoylation process, which is a D-lactylation (27, 28) (**Figure 3F**). Wang J et al. discovered that lactate accumulating from aerobic glycolysis in M1 macrophages leads to lactylation modification of PKM2 at K62, leading to PKM2 activation into its tetrameric form and reducing dimerization and nuclear translocation. This resulted in the suppression of the macrophage Warburg effect, decreased lactate production, and facilitated the transition of M1 macrophages to the M2 repair phenotype (101) (**Figure 3G**). The significance of this study lies in: (1) revealing a possible mechanism to regulate the inflammation/repair balance and the “lactate clock”: By limiting the rate of lactate production from macrophage aerobic glycolysis through negative feedback, it restricts the over-inflammation in M1 macrophages while delaying the activation of genes modified by histone lactylation to prevent inflammation from subsiding prematurely; (2) revealing that the PKM2 paradox in the Warburg effect exists not only during the pharmacological activation of PKM2, but also during the transition of macrophages from a pro-inflammatory to a reparative phenotype; (3) providing an explanation for why lactate failed to increase Arg-1 expression in M0 macrophages but did so in LPS-induced M1 macrophages in the Dichtl S et al. study: LPS stimulation upregulates PKM2 expression in macrophages, while lactate activates PKM2 through non-histone lactylation. Together, they work synergistically to inhibit glycolysis and increase Arg-1 expression. In addition, Yang K et al. found that exogenous lactate has deleterious effects on sepsis patients by mediating the lactylation and acetylation modifications of HMGB1 in macrophages, inducing the release of lactylated/acetylated HMGB1 from cells by exosomal secretion (102). As an important damage associated molecular pattern (DAMP) molecule, HMGB1 induces macrophage polarization toward the M1 phenotype while maintaining migratory capacity and some M2 characteristics (103). Subsequently, Du S et al. found a similar process in a hepatic ischemia-reperfusion injury model and found that heat shock protein A12A (HSPA12A) could exert its hepatoprotective effects by preventing M1 macrophage activation via inhibiting the lactylation and exosomal secretion of HMGB1 (104). HIF-1α can produce lactate by inducing aerobic glycolysis in macrophages in response to pro-inflammatory stimuli. Studies have demonstrated that the stability of HIF-1α can be regulated by a variety of post-translational modifications, such as acetylation, ubiquitination, and phosphorylation. Therefore, it is likely that a lactylation modification of HIF-1α occurs in response to pro-inflammatory stimuli. However, further research is still needed to determine whether lactylation exists in HIF-1α under pro-inflammatory stimuli, as well as the function of lactylation on HIF-1α and how it works (105, 106) (**Figure 3G**).

## 5 Role of lactylation in pathophysiological processes associated with macrophage metabolic reprogramming

### 5.1 Inflammation and repair

In macrophages, pro-inflammatory stimuli such as LPS and hypoxia enhance HIF-1α transcription by inducing the activation of NF-κB signaling. HIF-1α expression undergoes nuclear translocation and binds to HIF-1β to form a complex, which in turn binds to the hypoxia-responsive element (HRE) at the promoter of target genes. The inactive dimeric PKM2 also undergoes nuclear translocation and interacts with HIF-1α. They work together to regulate the expression of target genes, thereby inducing macrophage polarization to the M1 phenotype, which exerts pro-inflammatory effects in response to injurious stimuli and exacerbates the original inflammatory response (41, 107–109) (**Figure 3A**). In summary, M1 macrophages can increase aerobic glycolysis through at least the following mechanisms: (1) The NO generated during the arginine degradation by iNOS can impair mitochondrial OXPHOS, leading to an increase in macrophage aerobic glycolysis (18); (2) TLR can activate PI3K-AKT via BCAP, thereby enhancing aerobic glycolysis (99); (3) The mitochondrial network of M1 macrophages gradually breaks from elongated to fragmented after LPS stimulation. The expression and activity of PDH in the broken mitochondria are down-regulated, leading to inhibition of the pathway for the production of acetyl-CoA from pyruvate, thereby enhancing aerobic glycolysis (110). The accumulation of lactate produced by aerobic glycolysis in M1 macrophages triggers the endogenous “lactate clock” mechanism. This results in the delayed activation of the M2 characteristic genes such as Arg-1 and the M2 core transcription factor STAT6, both of which are regulated by H3K18 histone lactylation. Eventually, the M2 anti-inflammatory phenotype manifests in macrophages (21, 111). In addition, as mentioned previously, lactate accumulated in macrophages can exert an inhibitory effect on aerobic glycolysis through non-histone lactylation modifications: (1) Non-enzymatic lysine lactoylation of glycolytic enzymes can limit aerobic glycolysis by inhibiting enzyme activity (28); (2) Lactylation of PKM2 can both increase its activity and inhibit its nuclear translocation, thus reducing aerobic glycolysis (101). Therefore, lactylation may modulate the inflammatory injury/anti-inflammatory repair balance in tissues by affecting the metabolic reprogramming process in macrophages.

Additional research has demonstrated that H4K12 lactylation can mediate cellular inflammation in addition to H3K18la. Pan RY et al. found that H4K12la modification activated the transcription of LDHA, PKM2, and HIF-1α, thereby exacerbating the pro-inflammatory activation and dysfunction of microglia in Alzheimer’s disease through a positive feedback loop of glycolysis-H4K12la-PKM2 (112). Subsequently, in a study on the pathogenesis of diabetic cardiomyopathy, Ma XM et al. found that although lactate can induce both H3K18la and H4K12la in macrophages, it mainly mediated macrophage inflammatory responses and induced the expression of HIF-1α and inflammatory cytokines like IL-1β via H4K12la in a high-free fatty acid environment (113). The above studies suggest that different extracellular microenvironments may lead to histone lactylation at different sites, which in turn may exhibit different regulatory effects.

### 5.2 Fibrillation

Abnormal repair of tissues after inflammation can lead to the onset of fibrosis. Macrophages can be polarized to a pro-fibrotic phenotype in addition to an anti-inflammatory repair phenotype to modulate tissue fibrosis (114). In idiopathic pulmonary fibrosis (IPF), the pro-fibrotic phenotype of macrophages can be regulated by local tissue through histone lactylation. Cui H et al. demonstrated that myofibroblasts undergoing aerobic glycolysis secrete lactate into the extracellular environment, where myofibroblasts, alveolar macrophages, and lactate form fibrotic niches. Exogenous lactate can upregulate the expression of pro-fibrotic genes like Arg-1, PDGFA, THBS1, and VEGFA in alveolar macrophages through histone lactylation at the promoter, leading to the pro-fibrotic phenotype and accelerating the progression of pulmonary fibrosis (94, 115, 116). However, their research still has some limitations: (1) There isn’t any concrete proof that lactate-induced alterations in macrophage phenotype are involved in the regulation of pulmonary fibrosis; (2) It is yet unclear what functions histone lactylation serves in other cells throughout the fibrosis process, such as mesenchymal stem cells and alveolar epithelial cells.

### 5.3 Tumor

Previous research has demonstrated that tumor cells can shape TAM into an M2 immunosuppressive phenotype, where lactate is a key signaling molecule that controls macrophage metabolic reprogramming, hence suppressing immune function and promoting tumor growth. Lactate produced by tumor cells through glycolysis creates a lactate-enriched TME, which can affect macrophages recruited into the TME by different means such as being recognized by and binding to GPR132. Lactate can stabilize HIF-1α and induce the expression of M2 genes such as Arg-1, Fizz-1, Mgl-1, VEGF, and PPAR-γ, thereby inducing TAM polarize to an M2 immunosuppressive phenotype and promoting tumor growth, metastasis, and invasion (77, 82, 117) (**Figure 2**).

Recently discovered lactylation has provided hints on the molecular mechanism of the above regulatory roles of lactate. The study by Fang X et al. reached several conclusions: (1) Due to the presence of excess lactate in TME and the limited availability of glucose for macrophages, endogenous lactate produced by macrophage glycolysis is a non-essential factor in TAM polarization and tumor progression, instead, exogenous lactate produced by tumor cells is a key factor; (2) Exogenous lactate is transported into TAM via MCT1, which stabilizes HIF-1α and upregulates its expression by promoting histone lactylation at the H3K18 site of the promoter of M2 genes, such as Arg-1 and VEGF, in TAM, thereby inducing the polarization of TAM to an M2 phenotype; (3) The induction of macrophage polarization to an M2 phenotype by lactate and IL-4 is independent of the MPC-mediated metabolic pathway of mitochondrial respiration (118) (**Figure 2G**). Some literature has reported on the role of lactylation in immunosuppression and its contribution to tumor progression in various cancer diseases. Wang L et al. found that upregulating PCSK9 expression in a colon cancer model led to increased lactylation of macrophage proteins and a shift of TAM towards the M2 phenotype, suggesting a correlation between the two (119). Yang H et al. evaluated the lactylation levels of relevant prognostic genes in a gastric cancer model. Their findings showed that patients with high lactylation levels were associated with greater immune escape potential and lower immunotherapy response rates (120). Chaudagar K et al. identified that lactylation promotes TAM immunosuppressive phenotypic polarization and exerts pro-tumorigenic effects in a PTEN/p53-deficient prostate cancer model. They are also focusing on relevant anti-tumor therapies targeting the signaling pathways that inhibit the production of lactate by tumor cells (121, 122).

Research on the molecular mechanism of lactylation in promoting tumor progression and the development of anti-tumor targeting drugs based on it are still in progress. Therapies targeting the metabolic pathway and signaling pathway of lactate production, as well as the transport and recognition of lactate between tumor cells and macrophages, is a relatively novel research field in anti-tumor therapy research (123).

## 6 Concluding remarks

The metabolic reprogramming that occurs in macrophages during various pathophysiological processes in which they are involved has been extensively studied, yet there are still several questions at the molecular mechanism level that remain to be addressed. Lactylation is a newfound post-translational modification of proteins initially observed in macrophages. Its characteristic as mediated by the glycolytic product lactate suggests a novel connection between macrophage metabolic features and phenotypic transformation. This review summarizes the features of macrophage-associated metabolic reprogramming and the role of lactate metabolism therein. In addition, it discusses the molecular mechanisms identified so far for the regulation of macrophage metabolic reprogramming by lactylation modifications and their regulatory roles in related pathophysiological processes.

Although the mechanism by which lactylation regulates metabolic reprogramming in macrophages has been clinically translated and has yielded some results, there are still some issues that remain to be investigated at the molecular level: (1) The threshold lactate concentration for inducing lactylation as well as its specificity at tissue, cellular, and protein levels; (2) The mechanism by which the lactate concentration in the intranuclear microenvironment of the cell is regulated; (3) The specific mechanisms through which histone lactylation regulates the “lactate clock” and achieves the inflammatory damage/anti-inflammatory repair balance; (4) Whether histone lactylation can completely repolarize M1 macrophages into M2 macrophages remains questionable; (5) The types of genes regulated by histone lactylation and the mechanisms governing its gene specificity. However, given the prevalence of lactylation in organisms and its involvement in diverse pathophysiological processes, both basic research and clinical translation on lactylation regulating macrophage metabolic reprogramming offer promising directions for further exploration.

## Author contributions

BX: Conceptualization, Methodology, Writing – original draft. YL: Methodology, Writing – original draft. NL: Funding acquisition, Supervision, Writing – review & editing. QG: Funding acquisition, Supervision, Writing – review & editing.

## Glossary

**Table:**

ACO2	aconitase 2
Arg1	arginase 1
BCAP	B-cell adapter for PI3K
BMDM	bone-marrow-derived macrophage
CBP	cAMP response element-binding protein (CREB) binding protein
DAMP	damage associated molecular pattern
EMT	epithelial-mesenchymal transition
ERK	extracellular signal-regulated kinase
ETC	electron transport chain
F-1,6-BP	fructose 1,6-bisphosphate
F-2,6-BP	fructose 2,6-bisphosphate
F-6-P	fructose 6-phosphate
FAS	fatty acid synthesis
FMT	fibroblast-to-myofibroblast transition
FOXO1	forkhead box protein O1
G-6-P	glucose 6-phosphate
GLO	glyoxalase
GLUT1	glucose transporter protein 1
GSK3β	glycogen synthase kinase 3β
HDAC	histone deacetylase
HIF-1α	hypoxia-inducible factor-1α
HK	hexokinase
HMGB1	high mobility group box 1
HRE	hypoxia-responsive element
HSPA12A	heat shock protein A12A
iNOS	inducible nitric oxide synthase
IRF3	interferon regulatory factor 3
JAK	janus kinase
LDHA	lactate dehydrogenase A
LGSH	lactoylglutathione
MCT	monocarboxylate transporter
MGO	methylglyoxal
MPC	mitochondrial pyruvate carrier
mTORC1	mammalian target of rapamycin complex 1
mTORC2	mammalian target of rapamycin complex 2
NF-κB	nuclear factor kappa-light-chain-enhancer of activated B cells
NO	nitric oxide
OAT	ornithine aminotransferase
ODC	ornithine decarboxylase
OXPHOS	oxidative phosphorylation
PCSK9	proprotein convertase subtilisin/kexin type 9
PDH	pyruvate dehydrogenase
PDK1	pyruvate dehydrogenase kinase 1
PD-L1	programmed death-ligand 1
PFK1	phosphofructokinase 1
PFKFB	fructose-6-phosphate-2-kinase/fructose-2,6-bisphosphatase
PI3K	phosphoinositide 3-kinase
PK	pyruvate kinase
PKM2	pyruvate kinase M2
PPP	pentose phosphate pathway
PRR	pattern recognition receptor
ROS	reactive oxygen species
STAT3	signal transducer and activator of transcription 3
STAT6	signal transducer and activator of transcription 6
TAM	tumor-associated macrophages
TCA cycle	tricarboxylic acid cycle
TDE	tumor - derived exosome
TFEB	transcription factor EB
TLR	toll-like receptor
TME	tumor microenvironment
VEGF	vascular endothelial growth factor
